# PB.45. Correlation of perilesional tissue stiffness measured by ultrasound strain elastography with breast density

**DOI:** 10.1186/bcr3742

**Published:** 2014-11-03

**Authors:** A Di Batista, R English, R Sidebottom, R Adams, L Winter, A Noble, A Harris

**Affiliations:** 1 Oxford University, Oxford, UK; 2 Oxford University Hospitals NHS Trust, Oxford, UK

## Introduction

Over an 11-month period a series of patients with screen-detected breast masses were recruited with ethical approval into a BRC-funded study designed to evaluate the use of ultrasound elastography in the sizing of early breast cancer. We present results and imaging examples from 71 patients in whom BIRADS evaluation of breast density is correlated with elastography findings of the perilesional tissue.

## Methods

Ultrasound elastography was performed in addition to the standard assessment modalities using a Zonare z.one US unit. Elastography data were post processed and were classified in terms of the strain characteristics of the perilesional tissue into stiff or not stiff. Standard mammogram projections were evaluated by two experienced breast radiologists (RE and RA) for breast density using the BIRADS system of classification. Intraobserver (RE) and interobserver (RE, RA) consistency was evaluated. Histological diagnosis subsequently demonstrated invasive disease in 55 patients and noninvasive in 16 patients.

## Results

Intraobserver and interobserver consistency was 83% and 82% respectively. There was no case exceeding one category of difference. No correlation was demonstrated between perilesional tissue characteristics and background breast density. Sixty-two cases were classified as BIRADS 1 and 2, of whom 38 had perilesional stroma measures as stiff and 24 as not stiff. Nine cases were classified as BIRADS 3 or 4, of whom five had perilesional stroma measures as stiff and four as not stiff.

## Conclusion

These results suggest that perilesional tissue stiffness is not closely related to the underlying breast density.

